# Attributable Outcomes of Endemic *Clostridium difficile*–associated Disease in Nonsurgical Patients

**DOI:** 10.3201/eid1407.070867

**Published:** 2008-07

**Authors:** Erik R. Dubberke, Anne M. Butler, Kimberly A. Reske, Denis Agniel, Margaret A. Olsen, Gina D’Angelo, L. Clifford McDonald, Victoria J. Fraser

**Affiliations:** * Washington University School of Medicine, St. Louis, Missouri, USA; † Centers for Disease Control and Prevention, Atlanta, Georgia, USA

**Keywords:** Clostridium difficile, attributable mortality, outcomes, healthcare epidemiology, hospital-associated infections, research

## Abstract

Data are limited on the attributable outcomes of *Clostridium difficile*–associated disease (CDAD), particularly in CDAD-endemic settings. We conducted a retrospective cohort study of nonsurgical inpatients admitted for >48 hours in 2003 (N = 18,050). The adjusted hazard ratios for readmission (hazard ratio 2.19, 95% confidence interval [CI] 1.87–2.55) and deaths within 180 days (hazard ratio 1.23, 95% CI 1.03–1.46) were significantly different among CDAD case-patients and noncase patients. In a propensity score matched-pairs analysis that used a nested subset of the cohort (N = 706), attributable length of stay attributable to CDAD was 2.8 days, attributable readmission at 180 days was 19.3%, and attributable death at 180 days was 5.7%. CDAD patients were significantly more likely than controls to be discharged to a long-term-care facility or outside hospital. Even in a nonoutbreak setting, CDAD had a statistically significant negative impact on patient illness and death, and the impact of CDAD persisted beyond hospital discharge.

## Abstract 2

CDAD led to significantly worse outcomes in these patients.

*Clostridium difficile*–associated disease (CDAD) is an increasingly common cause of hospital-associated diarrhea (*1*,*2*). The emerging NAP1 strain of *C. difficile* has been associated with numerous outbreaks and appears to be more virulent than other endemic and epidemic *C. difficile* strains (*3*–*9*). Despite the increasing importance of this pathogen, few data exist on outcomes attributable to CDAD (*10*–*14*). The attributable mortality for CDAD has recently been estimated at 6.9% and 16.7% (*9*,*12*). However, these studies were performed during CDAD outbreaks caused by the NAP1 strain. Published estimates of CDAD-attributable deaths in disease-endemic settings are much lower (1.2%–1.5%) (*10*,*13*). Kyne et al. did not find endemic CDAD to be an independent predictor of death within 1 year of CDAD, but that study was relatively small (47 CDAD cases) (*11*). Thus, additional data with larger sample sizes are needed to determine outcomes associated with CDAD in nonoutbreak settings. With a large cohort of CDAD patients at a tertiary-care center, we evaluated CDAD outcomes including length of stay, hospital discharge status, time-to-readmission, and deaths in a CDAD-endemic setting.

## Methods

This study was conducted at Barnes-Jewish Hospital (BJH), a 1,250-bed, tertiary-care academic hospital in St. Louis, Missouri. Eligibility was limited to nonsurgical patients admitted for >48 hours from January 1 through December 31, 2003. Nonsurgical patients were defined as those without operating room costs. Surgical patients were excluded because of their heterogeneity. Specifically, risk factors for length of stay, readmission to the hospital, and death were different in this population compared with other hospitalized patients. Data were primarily collected from the hospital’s Medical Informatics database. The database was queried to collect patient demographics; admission and discharge dates; International Classification of Diseases, 9th edition, Clinical Modification (ICD-9-CM), diagnosis and procedure codes (Appendix); inpatient medication orders; vital signs; and laboratory results, including *C. difficile* toxin assay results. The Medical Informatics database was also queried to ascertain date of death. Patients without a death date in the database were screened for death by reviewing the Social Security Death Index.

For each patient, a modified APACHE II Acute Physiology Score (APS) was calculated to adjust for severity of illness (*15*). The APS was based on laboratory results and vital signs collected within 24 hours of admission. The score was modified because data for respiratory rates and Glasgow coma scores were unavailable electronically. In addition, the Charlson-Deyo method was used to classify co-existing conditions (*16*,*17*). Albumin levels within 24 hours of admission were collected and categorized into normal (>3.5 g/dL), low (2.5–3.5 g/dL), and very low (<2.5 g/dL). Multiple imputation methods were used to impute albumin levels for patients without recorded values (*18*). For CDAD case-patients, only medication and intensive-care unit exposures before the patient’s first positive stool toxin assay were included in analyses.

### Case Definition

CDAD case-patients were defined as inpatients with positive *C. difficile* stool toxin assays (TechLab, Blacksburg, VA, USA). The microbiology laboratory only performs toxin tests on unformed stool, so all patients with a positive result for toxin were considered case-patients. Both community-onset and hospital-onset CDAD case-patients were included in the analyses.

Analyses were performed on the full cohort and a nested case–control population. The first component was a retrospective cohort. For CDAD patients, the admission date when the patient’s CDAD was first identified was used as the index admission. For noncases with >1 admission during the study period, 1 admission was randomly selected as the index admission. The nested case–control population consisted of propensity score matched cases and controls from patients identified in the cohort.

### Cohort

#### Data Analysis

Survival was defined as the number of days from the index hospital admission until death. Survival was censored at 180 days. Time to readmission was calculated as the number of days between the index hospitalization discharge date and the date of the subsequent admission to BJH, if applicable. Days until readmission were censored at death or 180 days, whichever occurred first.

Fisher exact, χ^2^, and Mann-Whitney U tests were used to compare characteristics of patients with and without CDAD. Time-to-event methods were used to estimate the effect of CDAD on 180-day survival and time-to-readmission. Patients who died during the index hospitalization were excluded from the time-to-readmission analysis. Kaplan-Meier analysis was used to evaluate the unadjusted relationships between CDAD and time-to-event outcomes. Cox proportional hazards regression was used to estimate unadjusted and adjusted hazard ratios and 95% confidence intervals (CIs). All variables with biologic plausibility or p<0.15 in the univariate analysis were considered in the multivariable Cox regression analysis by using backward stepwise selection. Variables were sequentially removed from the final model, starting with the variable most weakly associated with the outcome. The significance of individual covariates was determined by using a Wald statistic of p<0.05. The proportional hazards assumption was verified by assessing the parallel nature of curves in log-log plots.

### Propensity Score Matched-Pairs Analysis

The second component of this study was a propensity score matched-pairs analysis of outcomes attributable to CDAD. This study design complemented the cohort by enabling analyses that could not be conducted in the entire cohort, specifically hospital discharge status, attributable length of stay, attributable time-to-readmission, and attributable death. Hospital discharge status could not be analyzed for the entire cohort because manual review of medical records was required to determine the discharge location of each patient. The large size of the cohort prohibited this analysis. In addition, survival and time-to-readmission estimates generated in the cohort analysis were validated in the matched-pairs analysis.

Cases and a subset of controls were selected from the primary cohort for the matched-pairs analysis. CDAD case-patients were matched to controls based on their propensity for CDAD to develop. Patient-specific probabilities of developing CDAD were predicted by a full logistic regression model adjusted for all variables suspected to impact the risk of developing CDAD (Appendix). Variables with p<0.05 in univariate analysis or biologic plausibility were included in the full logistic regression model. CDAD case-patients and controls were matched by a 1:1 ratio that used the nearest-neighbor method within calipers of 0.015 standard deviations (*19*). CDAD cases without an available nearest-neighbor control were excluded from the analysis. Chi-square, Fisher exact, and Mann Whitney U tests were used, as appropriate, to compare characteristics of CDAD case-patients and controls.

Medical records were reviewed for all CDAD case-patients and controls to determine hospital discharge location for each patient. Patients were categorized as being discharged to home, to a long-term-care facility, or to an outside hospital or dying in the hospital. Long-term-care facility was defined as a long-term-care facility, long-term acute-care facility/chronic ventilation facility, inpatient rehabilitation facility, skilled nursing facility, or nursing home. Outside hospital was defined as a non-BJH hospital or acute-care facility.

#### Data Analysis

Median length of stay was determined for CDAD case-patients and controls. The difference in median pairwise length of stay was compared with the Wilcoxon signed-rank test. Attributable length of stay was calculated as the median pairwise difference between CDAD case-patients and controls. Frequencies, adjusted odds ratios, and 95% CIs were calculated to determine if discharge location was associated with CDAD. CDAD-attributable 180-day readmission was calculated as the difference in readmission between CDAD case-patients and controls. Attributable deaths from 0–180 days, 0–60 days, and 61–180 days after admission were also calculated by using this method.

The primary survival endpoints of interest were death and readmission, which were both censored at 180 days or at death. Kaplan-Meier analyses, conducted by using log-rank tests, were used to determine relationships between the survival endpoints and CDAD. Cox proportional hazards regression stratified by matched-pairs was used to obtain hazard ratios and 95% CIs. Violation of the proportional hazards assumption was verified by the crossing nature of curves in the log-log plots. Therefore, we used an extended Cox regression model stratified by matched-pairs for the periods <60 days and >60 days. The breakpoint of 60 days was chosen because the graph of survival curves for CDAD case-patients and controls diverged at ≈60 days. Violation of the proportional hazards assumption was confirmed by the significance of the coefficient for the product term between CDAD and <60 days and >60 days (*20*).

All tests were 2-tailed, and p<0.05 was considered significant. Statistical analyses were performed with SPSS for Windows version 14.0 (SPSS, Inc., Chicago, IL, USA) and SAS version 9.1 (SAS Institute, Cary, NC, USA). The Washington University Human Studies Committee approved this project.

## Results

Among 18,050 nonsurgical inpatients admitted during the 1-year study period, 390 had CDAD and 17,660 did not. Selected patient characteristics of the cohort are summarized in Table 1. CDAD patients were significantly older (median 66.0 vs. 52.7 years, p<0.001) more likely to be men, and more likely to be Caucasian than were noncase-patients. CDAD case-patients had a higher severity of illness on admission than noncases, as indicated by the modified APS. CDAD patients were more likely to have a diagnosis of congestive heart failure, chronic obstructive pulmonary disease, cancer, leukemia or lymphoma, and metastatic solid tumors.

**Table 1:** Baseline characteristics of study cohort, *Clostridium difficile*–associated disease (N = 18,050)*

Characteristic	CDAD case-patients (n = 390), no. (%)	Non–case-patients (n = 17,663), no. (%)	p value†
Age, y			
<45	58 (15)	6,847 (39)	<0.001
45–65	132 (34)	5,187 (29)	0.06
>65	200 (51)	5,626 (32)	<0.001
Male sex	194 (50)	6,704 (38)	<0.001
White race	257 (66)	9,860 (56)	<0.001
Modified APS			
<2	77 (20)	6,687 (38)	<0.001
3–4	76 (20)	4,573 (26)	0.004
5–6	82 (21)	2,970 (17)	0.028
>7	155 (40)	3,430 (19)	<0.001
Liver disease			
Mild	5 (1)	204 (1)	0.77
Moderate to severe	6 (2)	209 (1)	0.47
Diabetes without chronic complications	70 (18)	2,718 (15)	0.17
Diabetes with chronic complications	15 (4)	416 (2)	0.06
Myocardial infarction	26 (7)	1466 (8)	0.25
Congestive heart failure	97 (25)	2,562 (15)	<0.001
Cerebral vascular disease	16 (4)	882 (5)	0.42
Chronic obstructive pulmonary disease	90 (23)	2,564 (15)	<0.001
Rheumatologic/collagen vascular disease	11 (3)	361 (2)	0.29
Peptic ulcer disease	5 (1)	279 (2)	0.64
Cancer, excluding leukemia or lymphoma	45 (12)	1,283 (7)	0.001
Leukemia or lymphoma	69 (18)	567 (3)	<0.001
Metastatic solid tumor	33 (9)	936 (5)	0.01
HIV/AIDS	5 (1)	209 (1)	0.81
Paraplegia or hemiplegia	8 (2)	223 (1)	0.17

*CDAD, *Clostridium difficile*–associated disease; APS, Acute Physiology Score. † Fisher exact test, χ^2^ test.

Of 17,492 patients alive at the index hospitalization discharge, 4,207 (24%) were readmitted to BJH within 180 days. Fifty-two percent of CDAD patients were readmitted within 180 days versus 23% of noncases (log-rank p<0.001). Univariate and multivariable Cox regression results for time to readmission are presented in Table 2. The adjusted hazard ratio (HR) for readmission within 180 days was significantly higher for CDAD case-patients than noncases (HR 2.19, 95% CI 1.87–2.55) (Table 2).

**Table 2:** Cox proportional hazards estimate of readmission at 180 d in *Clostridium difficile*–associated disease (CDAD) study cohort  (N = 17,492; 4m207 readmissions, 13,285 censored)*†

Variable	Univariate hazard ratio‡ (95% CI)	Multivariable hazard ratio ‡ (95% CI)
CDAD	3.09 (2.95–3.23)	2.19 (1.87–2.55)
Male sex	1.42 (1.40–1.45)	1.11 (1.05–1.19)
White race	1.26 (1.23–1.28)	1.06 (1.00–1.13)
Modified APS		
<2	Reference	Reference
3–4	1.15 (1.12–1.18)	1.10 (1.02–1.20)
5–6	1.39 (1.35–1.43)	1.24 (1.13–1.35)
>7	1.84 (1.80–1.89)	1.50 (1.37–1.64)
Albumin, g/dL§		
>3.5	Reference	Reference
2.5–3.5	1.05 (1.03–1.08)	0.99 (0.92–1.08)
<2.5	1.03 (0.99–1.07)	0.95 (0.80–1.14)
Liver disease		
None	Reference	Reference
Mild	1.80 (1.67–1.94)	1.44 (1.12–1.83)
Moderate to severe	1.79 (1.65–1.94)	1.48 (1.13–1.93)
Diabetes with chronic complications	1.89 (1.80–1.99)	1.53 (1.30–1.80)
Diabetes without chronic complications	1.29 (1.26–1.32)	1.10 (1.02–1.19)
Congestive heart failure	1.60 (1.56–1.64)	1.34 (1.23–1.45)
Cerebrovascular disease	0.77 (0.74–0.81)	0.74 (0.63–0.87)
Cancer, excluding leukemia or lymphoma	2.75 (2.67–2.83)	1.90 (1.70–2.13)
Leukemia or lymphoma	2.31 (2.18–2.45)	1.84 (1.52–2.23)
Metastatic solid tumor	2.81 (2.71–2.91)	1.66 (1.46–1.90)
HIV/AIDS	1.74 (1.62–1.87)	1.74 (1.38–2.19)
ICU admission	1.06 (1.03–1.09)	0.84 (0.76–0.93)

*CI, confidence interval; APS, Acute Physiology Score; ICU, intensive care unit. †The analysis excluded 558 patients who died during the index hospital admission. Nonsignificant variables considered in the model included mechanical ventilation, paraplegia/hemiplegia, chronic obstructive pulmonary disease, myocardial infarction, rheumatologic/collagen vascular disease, and peptic ulcer disease. ‡Hazard ratios also adjusted for categorical age (<20, 20–24, 25–29, 30–34, 35–39, 40–44, 45–49, 50–54, 55–59, 60–64, 65–69, 70–74, 75–79, 80–84, 85–89, 90–94, >95 y). §7,610 (42%) patients were missing albumin values. Values were imputed by using multiple imputation methods.

**Figure 1:**
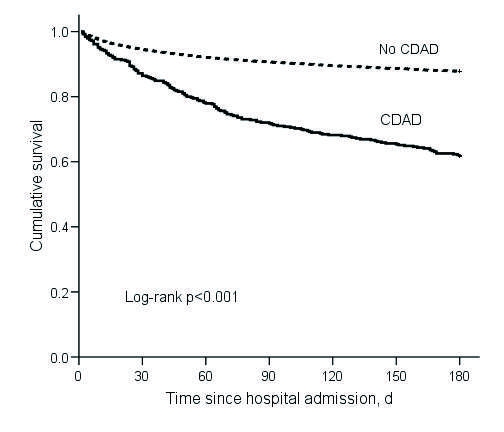
Kaplan-Meier survival estimates for cohort (N = 18,050). CDAD, *Clostridium difficile*–associated disease.

By 180 days after hospital admission, 149 (38%) of 390 CDAD case-patients and 2,150 (12%) 17,660 noncase-patients had died. In the Kaplan-Meier analysis, the mortality rate was significantly higher for CDAD case-patients than noncases (log rank p<0.001) (Figure 1). Unadjusted and adjusted Cox regression results for death within 180 days of admission (“180-day mortality”) are presented in Table 3. The adjusted hazard ratio for 180-day mortality was significantly higher for CDAD case-patients than noncase patients (HR 1.23, 95% CI 1.03–1.46) (Table 3).

**Table 3:** Cox proportional hazards estimate of deaths from *Clostridium difficile*–associated disease (CDAD) at 180 d in study cohort (N = 18,050; 2,299 deaths, 15,751 censored)*†

Variable	Univariate hazard ratio‡ (95% CI)	Multivariable hazard ratio‡ (95% CI)
CDAD	3.55 (3.37–3.75)	1.23 (1.03–1.46)
Male sex	1.73 (1.68–1.77)	1.17 (1.08–1.27)
White race	1.65 (1.61–1.70)	1.22 (1.11–1.33)
Modified APS		
<2	Reference	Reference
3–4	1.41 (1.36–1.47)	1.09 (0.96–1.24)
5–6	2.09 (2.00–2.17)	1.30 (1.14–1.49)
>7	4.11 (3.97–4.25)	1.65 (1.46–1.87)
Albumin, g/dL§		
>3.5	Reference	Reference
2.5–3.5	2.12 (1.90–2.36)	1.62 (1.45–1.82)
<2.5	4.77 (3.91–5.81)	2.93 (2.52–3.42)
Liver disease		
None	Reference	Reference
Mild	3.08 (2.86–3.33)	2.37 (1.85–3.04)
Moderate to severe	5.50 (5.17–5.85)	3.76 (3.05–4.64)
Diabetes with chronic complications	1.47 (1.37–1.58)	1.49 (1.18–1.88)
Congestive heart failure	1.85 (1.80–1.91)	1.28 (1.15–1.42)
Cerebrovascular disease	1.68 (1.60–1.76)	1.62 (1.37–1.92)
Cancer, excluding leukemia or lymphoma	6.42 (6.24–6.61)	2.44 (2.15–2.76)
Leukemia or lymphoma	3.17 (2.99–3.38)	4.92 (3.98–6.08)
Metastatic solid tumor	8.82 (8.57–9.09)	4.41 (3.87–5.03)
HIV/AIDS	1.77 (1.62–1.95)	2.88 (2.12–3.91)
Paraplegia/ hemiplegia	1.75 (1.60–1.92)	1.53 (1.12–2.07)
Mechanical ventilation	6.39 (6.18–6.62)	3.17 (2.71–3.71)
ICU admission	3.08 (2.99–3.17)	1.31 (1.14–1.50)

*CI, confidence interval; APS, Acute Physiology Score; ICU, intensive care unit. †Nonsignificant variables considered in the model included diabetes without chronic complications, chronic obstructive pulmonary disease, myocardial infarction, rheumatologic/collagen vascular disease and peptic ulcer disease. Of 2,299 people who died within 180 d of admission, 1,565 (68%) deaths were identified by means of the hospital Medical Informatics database and 734 (32%) were identified with the Social Security Death Index. ‡Hazard ratios also adjusted for categorical age (<20, 20–24, 25–29, 30–34, 35–39, 40–44, 45–49, 50–54, 55–59, 60–64, 65–69, 70–74, 75–79, 80–84, 85–89, 90–94, ≥95 y). §7,525 (43%) patients were missing albumin values. Values were imputed by using multiple imputation methods.

The propensity score matched-pairs analysis included 353 CDAD cases and 353 controls (N = 706). There were no significant differences between the matched cases and controls after correcting for multiple testing with the Bonferroni procedure. Thirty-seven CDAD case-patients were dropped because a nearest-neighbor control was not available. Unmatched CDAD patients had significantly higher modified APS (median = 7.0 vs. 5.0, p<0.001), longer median length of stay (13.6 days vs. 9.6 days, p = 0.01), and higher percentage of deaths at 180 days (59% vs. 36%, p = 0.01) than matched case-patients.

In the matched-pairs analysis, median length of stay was 9.6 days for CDAD patients compared with 5.8 days for controls, and the increased attributable length of stay for CDAD patients was 2.8 days (Wilcoxon signed-rank p<0.001). Among the 706 patients in the matched-pairs analysis, 445 (63%) were discharged to home and 188 (27%) were discharged to a long-term-care facility. Only 7 (1%) patients were discharged to an outside hospital; therefore, these patients were combined with patients discharged to a long-term-care facility in the analysis. CDAD patients were significantly more likely than controls to be discharged to a long-term-care facility or outside hospital (32% vs. 23%, odds ratio 1.62, 95% CI 1.15–2.28, McNemar p = 0.01).

**Figure 2:**
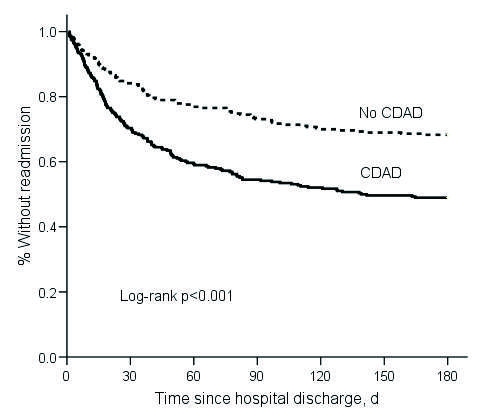
Kaplan-Meier estimates of time until hospital readmission for matched pairs (n = 580). CDAD, *Clostridium difficile*–associated disease.

Among 290 matched-pairs with both patient and control alive at index hospitalization discharge, 148 CDAD patients were readmitted to BJH within 180 days compared with 92 controls, for an attributable readmission of 19.3% (11.4%–27.2%). In the Kaplan-Meier and Cox model analyses, CDAD patients were significantly more likely than controls to be readmitted to the hospital within 180 days (Figure 2, Table 4).

**Table 4:** Cox proportional hazards model estimates of readmission and death of matched-pairs analysis, *Clostridium difficile*–associated disease (CDAD)*

Variable	CDAD case-patients, no. (%)	Controls, no. (%)	Hazard ratio (95% CI)
Readmitted within 180 d†	148 (51.0)	92 (31.7)	2.17 (1.59–2.95)
Deaths at 180 d‡	127 (36.0)	107 (30.3)	1.22 (0.92–1.61)
Deaths at 0–60 d‡	72 (20.4)	75 (21.2)	0.96 (0.54–1.70)
Deaths at 61–180 d‡	55 (15.6)	32 (9.1)	2.00 (1.47–2.72)

*CI, confidence interval. †n = 290 matched pairs; 63 matched pairs were excluded because one or both patients in the pair died during the index hospital admission. ‡n = 353 matched pairs.

**Figure 3:**
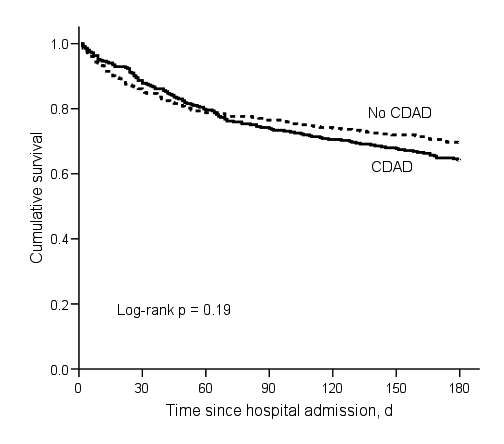
Kaplan-Meier survival estimates for matched pairs (n = 706). CDAD, *Clostridium difficile*–associated disease.

By 180 days after hospital admission, 127 CDAD patients died compared with 107 controls, for an attributable mortality of 5.7% (95% CI –1.3%–12.6%). Although CDAD case-patients were no more likely than controls to die within 60 days of hospital admission, a divergence in survival between CDAD case-patients and controls began 60 days after hospital admission (Figure 3, Table 4). The HR for death from 61–180 days was significantly higher for CDAD patients than controls (HR 2.00, 95% CI 1.47–2.72) (Table 4). Among 223 matched-pairs with both case-patients and controls alive after day 60, 19.7% of CDAD patients and 12.6% of controls died within 180 days for an attributable mortality between 61–180 days of 7.2% (95% CI 0.4%–14.0%).

## Discussion

The results of this study indicate that CDAD is a major contributor to death even in nonoutbreak settings. In this CDAD-endemic setting, the disease was associated with a 23% higher hazard of death within 180 days after hospital admission in the multivariable cohort analysis and a 7.2% attributable mortality 61–180 days after hospital admission in the matched-pairs analysis. Historically, endemic CDAD has been reported to be associated with minimal increased risk in mortality although NAP1 strain CDAD outbreaks have been associated with much higher attributable mortality (*10*,*11*,*13*). Two studies of CDAD in endemic settings reported 1.2%–1.5% inhospital mortality rates from CDAD (*10*,*13*). Using a multivariable Cox proportional hazards model, Kyne et al. found no association between CDAD and 1-year mortality, although that study was quite small (47 CDAD patients) (*11*). In contrast, several studies have identified increased deaths associated with outbreaks of the NAP1 strain. Pepin et al. estimated the 1-year attributable mortality of CDAD during an outbreak with the NAP1 strain to be 16.7% (*9*). Hubert et al. reported that CDAD was the attributable or contributive cause of death in 8.4% of patients infected with a strain of *C. difficile* that had the binary toxin and *tcdC* deletion (*21*). Loo et al. found CDAD to be the attributable cause of death within 30 days in 6.9% of CDAD patients and suspected that CDAD contributed to death in another 7.5% of CDAD patients (*12*). The estimate of 6.9% attributable mortality, however, was determined through chart review, not through multivariable analyses, and medical chart review may not be an adequate method to determine attributable mortality because of subjectivity (*22*).

Although the 5.7% 180-day attributable mortality determined in the propensity score matched-pairs analysis in our study was not statistically significant, the estimate is substantially higher than estimates reported from other CDAD-endemic settings. The attributable mortality we report is more consistent with estimates from outbreaks of the NAP1 strain and is likely clinically significant. The NAP1 strain was first identified at BJH during 2005, but the strain may have been present during the study period (*23*). During the years 2000–2006 at BJH, there were no apparent increases in hospital-onset CDAD incidence rates or severity of CDAD (as measured by the number of colectomies per CDAD case per year and by the percentage of patients with CDAD who died during hospitalization) (data not shown). Thus, the high attributable mortality found in this study has important implications for patients: CDAD remains an important cause of patient death even in a CDAD-endemic setting.

Our study showed that CDAD had a delayed impact on death. In the matched-pairs analysis, the divergence in survival between CDAD cases-patients and controls did not begin until >60 days after hospital admission. Within 60 days of admission, survival was not significantly different between CDAD patients and controls, when all but 4 (1%) patients had been discharged from the hospital. This finding is consistent with those of 2 recent nested matched case–control studies in nonoutbreak settings, in which no significant excess deaths were reported after 30 days (*24*) or at discharge (*25*). Although CDAD can be acutely life-threatening, delayed death caused by CDAD may not be easily recognized as related to the initial CDAD episode. CDAD may contribute to a decline in patient function and overall illness over time, ultimately leading to death in many patients.

The results of the time-to-readmission and discharge location analyses further emphasize the negative impact of CDAD. CDAD patients were more than twice as likely to be readmitted to BJH within 180 days compared with controls. This finding is consistent with our prior findings that CDAD contributes to an increase in hospital costs extending out to at least 180 days (*26*). CDAD patients were also significantly more likely to be discharged to a long-term-care facility or outside hospital. Few data are available on the health of CDAD patients after hospital discharge, and future studies following CDAD patients as outpatients over an extended period are needed.

Data on the excess length of hospital stay attributable to CDAD are limited. Wilcox et al. found that CDAD patients stayed in the hospital, on average, 21.3 days longer than non-CDAD patients; however, the attributable length of stay was not calculated (*14*). O’Brien et al. reported that the mean increase in hospitalization among CDAD patients was 2.9 days (*27*). Kyne et al. calculated the attributable length of stay at 3.6 days (*11*), which was comparable to the attributable length of stay estimate found in our study (2.8 days).

Our study has several limitations, including the retrospective study design. Use of electronic data from the hospital’s Medical Informatics database has limitations, although use of these data made analysis of such a large cohort feasible. Differences seen in observational studies may be due to unmeasured confounders. We attempted to address this issue by using 2 methods to control for confounding: multivariable regression analyses and propensity score matched-pairs analyses. As evident from the Kaplan-Meier mortality analyses, the matched-pairs population is a more homogeneous population than the cohort. This design allows more precise effect estimation because the association between CDAD and the propensity score variables among the study participants is eliminated. A strength of the multivariable regression analyses is the use of all available data in the cohort. In the propensity score matched-pairs analyses, 37 CDAD cases were excluded because of lack of a suitable control. Unmatched case-patients were more severely ill than matched case-patients, and their exclusion is a limitation of the propensity-score matched-pairs analyses. In the time-to-readmission analyses, we were unable to identify readmissions to hospitals other than our institution. Finally, surgical patients were excluded from these analyses. Because of this exclusion, the most severely ill CDAD patients requiring colectomies (n = 3) were not represented in the dataset. The absence of these patients, as well as the 37 unmatched case-patients, may have resulted in estimates of attributable length of stay and death that are biased low.

Data on attributable outcomes associated with CDAD are scarce. As previously mentioned, some data on attributable mortality and length of stay exist; however, these findings are limited by lack of adequate controls, small sample size, or outbreak settings. Our study provided detailed analysis on the effect of CDAD on time-to-readmission. Another key strength of this study is the combination of 2 analytical methods: Cox proportional hazards regression in the primary cohort and propensity score matched-pairs analysis. Mortality and time-to-readmission analyses, which were conducted in both the cohort and matched-pairs populations, had remarkably similar results. The results of this study suggest that endemic CDAD can lead to significantly poorer patient outcomes, including increased hospital length of stay, death, risk for admission to a long-term-care facility, and risk for hospital readmission. Even when the most severe CDAD cases are not considered, the detrimental effect of CDAD on patient health appears to extend beyond hospital discharge. Although prospective validation of these findings is needed, proper allocation of healthcare resources toward prevention of this infection is necessary to prevent further illness and death attributable to CDAD.

## Supplementary Material

Appendix Details on ICD-9-CM Codes and Creation of the Propensity Score for Clostridium difficile-associated disease
