# Supplementary material for: Attributable Outcomes of Endemic Clostridium difficile–associated Disease in Nonsurgical Patients
Source: Emerg Infect Dis. 2008 Jul;14(7):1031–8. doi: 10.3201/eid1407.070867 (PMC2600322; doi:10.3201/eid1407.070867)
Supplement: Appendix — Details on ICD-9-CM Codes and Creation of the Propensity Score for Clostridium difficile-associated disease [file 07-0867_appT-s1.pdf]

Appendix.

Details on ICD-9-CM Codes and Creation of the Propensity Score for *Clostridium difficile*–associated disease

Details on ICD-9-CM Codes

The International Classification of Diseases, 9th edition, Clinical Modification (ICD-9-CM) system of classifying hospital discharge diagnoses and procedures is used throughout the United States. In this study, all coexisting conditions were identified by ICD-9-CM diagnoses codes, and procedures were identified by ICD-9-CM procedure codes. The Deyo adaptation of the Charlson Comorbidity Index was used to identify and classify coexisting conditions (*1,2*). Medical coders assign ICD-9-CM diagnoses and procedure codes after reviewing medical record documentation.

Creation of the Propensity Score

The propensity score predicts the probability of developing *Clostridium difficile*–associated disease (CDAD) (from 0 to 1) for each patient in the dataset, with a higher score indicating a higher probability of CDAD's developing. By matching CDAD case-patients to controls based on propensity score, the association between CDAD and multiple outcomes can be assessed with adjustment for confounding. To calculate the propensity score, all known variables suspected to affect the development of CDAD were included as independent variables in a multivariable logistic regression analysis. Additional variables that significantly affected hospital length of stay or death were included as well. Patient-specific probabilities were generated by a multivariable logistic regression model with CDAD as the dependent variable. The independent variables are presented in the online Appendix Tables 1 and 2.

Some coexisting conditions were classified by the Deyo adaptation of the Charlson Comorbidity Index (*1,2*). For each patient, a modified APACHE II Acute Physiology Score was calculated to adjust for severity of illness (*3*).

Appendix Table 1. ICD-9-CM codes used to create independent variables included in the

propensity score*

Variable	ICD-9-CM code
Comorbidities identified by ICD-9-CM diagnoses codes	
Congestive heart failure (Deyo)	428.0–428.9
Cerebrovascular disease (Deyo)	430–438.0
Moderate or severe liver disease (Deyo)	572.2–572.8
Any malignancy, excluding leukemia and lymphoma (Deyo)	140.0–172.9<br>174.0–195.8
Leukemia or lymphoma (Deyo)	200.0–208.91
Metastatic solid tumor (Deyo)	196.0–199.1
Deep venous thrombosis or pulmonary embolism	415.1–415.11, 453.40–453.9
Cardiac arrest	427.5
Atrial fibrillation	427.31
Hypertension	401.0–401.9
Cystic fibrosis	277.0–277.09
Pleurisy, pneumothorax, or pulmonary collapse	510.0–512.8, 518.0–518.2
Acute renal failure	584.5–584.9, 586
Urinary tract infection or pyelonephritis	590.00–590.80, 599.0
Anemia	280.0–282.3, 282.8–285.9
Neutropenia	288.0
Convulsions	780.31–780.39
Depression	311
Schizophrenia	295.00–295.95
Episodic mood disorders	296.00–296.99
Adverse drug event or drug overdose	960.0–979.9
Vaginal delivery	650, V27.0
Early or threatened labor	644.00–644.21
Procedures identified by ICD-9-CM procedure codes	
Insertion, repair, or removal of pacemaker or defibrillator	00.50–00.54, 37.61–37.99
Angioplasty or stent placement (coronary and noncoronary)	00.55–00.65, 36.01–36.09, 39.50
Cardiac stress tests, pacemaker, and defibrillator checks	89.41–89.49
Aneurysm repair	37.32, 39.51–39.52, 39.71–39.79
Central venous catheter placement	38.93
Minor surgery or procedures on colon or small intestine	46.0–46.99
G-tube placement	43.0–43.19
Hemodialysis	39.95
Chemotherapy	99.25
Cesarean section	74.0–74.4, 74.99
Medical or surgical complication	996.0–996.59, 996.7–997.3, 998.0–999.9, E87.00–E87.99
Psychiatric somatotherapy	94.21–94.29

*ICD-9, International Classification of Diseases, 9th edition, Clinical Modification.

Appendix Table 2. Other independent variables included in the propensity score, *Clostridium difficile*–associated disease

Demographic variables
Age
Race (white or nonwhite)
Sex
Medication variables
Treatment with gastric acid suppressors
Treatment with antidiarrheals
Treatment with laxatives
Treatment with narcotics
No. days receiving antifungal
No. days receiving antiviral
No. days receiving amoxicillin/ampicillin
No. days receiving metronidazole
No. days receiving fluoroquinolone
No. days receiving intravenous vancomycin
No. days receiving macrolide
No. days receiving cephalosporins
No. days receiving clindamycin
Additional variables

Modified Acute Physiology Score (*3*)
Albumin (>3.5, 2.5–3.5, and <2.5 g/dL)
No. procedures performed
Admission to chronic ventilation floor
No. days in intensive care unit
Hospital admission in previous 60 days
Sum *Clostridium difficile*–associated disease pressure (*4*)

Appendix References

1. Charlson ME, Pompei P, Ales KL, MacKenzie CR. A new method of classifying prognostic comorbidity in longitudinal studies: development and validation. J Chronic Dis. 1987;40:373–83.

2. Deyo RA, Cherkin DC, Ciol MA. Adapting a clinical comorbidity index for use with ICD-9-CM administrative databases. J Clin Epidemiol. 1992;45:613–9.

3. Knaus WA, Draper EA, Wagner DP, Zimmerman JE. APACHE II: a severity of disease classification system. Crit Care Med. 1985;13:818–29.

4. Dubberke ER, Reske KA, Olsen MA, McMullen KM, Mayfield JL, McDonald LC, et al. Evaluation of "CDAD pressure" as a risk factor for *Clostridium difficile*-associated disease. Arch Intern Med. 2007;167:1092–7.
